# Kynurenic acid modulates experimentally induced inflammation in the trigeminal ganglion

**DOI:** 10.1186/s10194-015-0581-x

**Published:** 2015-12-01

**Authors:** A. Csáti, L. Edvinsson, L. Vécsei, J. Toldi, F. Fülöp, J. Tajti, K. Warfvinge

**Affiliations:** Department of Neurology, University of Szeged, Semmelweis 6, H-6725 Szeged, Hungary; Department of Medicine, Institute of Clinical Sciences, Lund University, Sölvegatan 17, SE 221 84 Lund, Sweden; MTA-SZTE Neuroscience Research Group, Szeged, Hungary; Department of Physiology, Anatomy and Neuroscience, University of Szeged, Szeged, Hungary; Institute of Pharmaceutical Chemistry and Research Group for Stereochemistry, Hungarian Academy of Sciences, University of Szeged, Eotvos u. 6, 6720, Szeged, Hungary

**Keywords:** Temporomandibular joint inflammation, Trigeminal ganglion, Kynurenic acid, Kynurenic acid amide 2, Cell signaling pathways

## Abstract

**Background:**

The trigeminal ganglion (TG) plays a central role in cranial pain. Administration of complete Freund’s adjuvant (CFA) into the temporomandibular joint (TMJ) elicits activation of TG. Kynurenic acid (KYNA) is an endogenous excitatory amino acid receptor blocker, which may have an anti-inflammatory effect. We hypothesize that KYNA may reduce CFA-induced activation within the TG.

**Methods:**

A local inflammation was induced by administration of CFA into the TMJ in rats. KYNA and kynurenic acid amide 2 (KYNAA2) were intraperitoneally administered. We investigated changes of mitogen-activated protein kinases (MAPKs as ERK1/2, p38 and SAPK/JNK), NF-κB, CaMKII and DREAM, in addition to calcitonin gene-related peptide (CGRP) and its receptor components calcitonin receptor-like receptor (CLR) and receptor activity-modifying protein 1 (RAMP1) in the TG, with immunohistochemistry and Western blot at 2 and 10 days post-CFA injection.

**Results:**

We showed CFA-induces increases in pERK1/2, pp38, CaMKII, NF-κB and DREAM immunohistochemistry after 2 and 10 days. KYNAA2 displayed stronger effects on MAPKs than KYNA. Increased expression of CaMKII, NF-κB and DREAM were found in the neurons. Western blot showed significantly increase in pERK expression at 10 days post-CFA, which decreased after 10 days of KYNA treatment. Two days post-CFA, a significantly increase in pp38 expression was found, which decreased after 2 days of KYNA and KYNAA2 treatment.

**Conclusions:**

The CFA-induced inflammatory model for the TG activation provided a time-related expression of MAPK (pERK1/2, pp38) and NF-κB. It involves both the neuronal and glial activation, which points to possible neuron-glia interactions during this process. The administration of the endogenous NMDA-receptor antagonists, KYNA and its derivative KYNAA2, resulted in the inhibition of the induced signaling system of the TG, which further points the importance of the glutamate receptors in this mechanism.

**Electronic supplementary material:**

The online version of this article (doi:10.1186/s10194-015-0581-x) contains supplementary material, which is available to authorized users.

## Background

The trigeminal ganglion (TG) through the mandibular branch (V3) of the trigeminal nerve projects sensory innervation to the temporomandibular joint (TMJ) capsule. TG contains mainly two populations of cells: neurons of different size and satellite glial cells (SGCs). The neurons project both in central and peripheral direction with unmyelinated C-fibers and thinly myelinated Aδ-fibers to transmit sensory information of different modalities. The functional importance of SGCs in sensory ganglia is less well understood, but neuron-glia interaction is presumed as the SGCs may process and transmit chemical signals, and regulate their microenvironment [1–3].

Numerous studies have used chemical stimulation of peripheral nerve branches with Complete Freund’s adjuvant (CFA) as an inflammatory model for investigating changes in the levels of various messenger and mediator molecules within the TG and in the CNS after experimentally induced TMJ inflammation [4–6]. It has been shown that the mitogen-activated protein kinase (MAPK) system can be activated by local application of CFA [7–9]. The MAPKs, a family of serine/threonine kinases, are involved in cellular responses to external signals such as pain [10, 11], growth factors, stress and inflammatory mediators [12–15]. The three major MAPKs are p38, extracellular signal-regulated kinase 1/2 (ERK1/2) and stress-activated protein kinase/c-jun N-terminal kinase (SAPK/JNK). These are located at the end of a dynamic chain of kinases [16] and are able to phosphorylate several downstream targets. The initial stimulus for this cascade varies between the MAPKs. ERK1/2 is often activated by growth factors [12], while p38 and SAPK/JNK are stress-activated protein kinases, which respond to cellular stress and inflammatory cytokines [14, 15, 17]. Activation of MAPKs can result in e.g. apoptosis, differentiation and proliferation [18].

Furthermore, it is well known that cytokines can activate different signaling pathways, including MAPKs [19]. Activation of MAPKs initiates the induction of nuclear factor kappa B (NF-κB) [20, 21], a key factor in the regulation of transcription of genes including inducible nitric oxide (NO) synthase, cyclooxygenase-2, tumor necrosis factor-α (TNF-α), interleukin-1β and interleukin-6 [22, 23]. Moreover, calcium calmodulin-dependent protein kinase II (CaMKII) plays a role in nociception and pain transmission in the trigeminal nucleus caudalis [24–26]. Intracellular calcium also modulates the nuclear translocation of the downstream regulatory element antagonist modulator (DREAM), which plays a role in endogenous responses to pain [27, 28]. DREAM −/− knock-out mice display reduced pain behavior in models of acute thermal, mechanical and visceral pain, and in chronic neuropathic and inflammatory pain [29].

Calcitonin gene-related peptide (CGRP) plays a key role in migraine pathophysiology [30]. CGRP is stored in TG small-medium sized neurons and its receptor components calcitonin receptor-like receptor (CLR) and receptor activity-modifying protein 1 (RAMP1) in large neurons and glial cells [31]. Upon activation of the TG, CGRP is released. Blocking the CGRP signaling with CGRP receptor antagonists has opened a possible option in migraine treatment [32].

Kynurenic acid (KYNA), a metabolite of the kynurenine pathway is produced by astrocytes and neurons [33]. It prevents neuronal loss following excitotoxic and ischaemic induced neuronal injuries [34]. Previously it has been shown that a series of kynurenic acid amines were found to be N-metyl-D-aspartat (NMDA) receptor NR2B subunit antagonists. In the study, a newly synthesized KYNA analogue, kynurenic acid amide 2 (KYNAA2, *N*-(2-*N*,*N*-dimethylaminoethyl)-4-oxo-1*H*-quinoline-2-carboxamide hydrochloride) was tested and shown to have neuroprotective potential [35]. Furthermore, KYNA also acts on glutamatergic and nicotinergic neurotransmission [36–40].

The aim of this study was to investigate the time-related changes of the cell signaling pathways in the TG following *in vivo* application of CFA into the TMJ. Because glutamate is a co-transmitter in the trigeminal system, we hypothesize that KYNA and derivative KYNAA2 might inhibit intracellular signaling pathways during CFA induced activation.

## Methods

### Animals

Adult male Sprague–Dawley rats (200-300 g, *n =* 6 in each group for immunohistochemistry and Western blot) were used in the experiments. Animals were raised and maintained under standard laboratory conditions. The study followed the guidelines of the European Communities Council (86/609/ECC) and approved by the Ethics Committee of The Faculty of Medicine, University of Szeged (I-74-12/2012, XI./352/2012).

The CFA injection was performed with the antero-superior technique, established by Kameoka et al. [41], into the right upper TMJ. Briefly, the TMJ region was identified by palpation. A 27-gauge needle was advanced into the TMJ space along the superomedial border of the zygomatic arch until it reached the condyle. This method of administration to the TMJ has been shown to be very accurate according to arthrographic CT of the joint, using contrast media [41].

The CFA injection (0.05 ml) consisted of 300 μg heat killed and dried *Mycobacterium tuberculosis*, paraffin oil and mannide monooleate [42], (Sigma-Aldrich, St. Louis, MO, USA), diluted in saline (oil:saline 1:1), creating an emulsion. One hour before CFA injection, the rats were pretreated with KYNA (Sigma-Aldrich, St. Louis, MO, USA) or the KYNAA2 in an equimolar, 300 mg/kg bodyweight dose (diluted to 2 ml, pH 7.4), intraperitoneally [26, 43, 44]. KYNA and KYNAA2 treatments were then carried out every 12 h for 48 h (termed 2 days post-CFA injection). Zhou et al. [45] demonstrated that the Fos protein, a marker of neuronal activation, peaked at 24–48 h after CFA injection into the TMJ. Consequently, we chose 48 h as survival time in order to evaluate the effect of the different treatments. In addition, KYNA treatment was applied for 10 days (termed 10 days post-CFA injection) to demonstrate possible long-term effects.

The control inflammatory groups received only the CFA injection and these rats were terminated at 2 and 10 days post-injection according to Zhou et al. [45]. Healthy, untreated rats were used as normal controls (termed Fresh). Before TMJ injections animals were deeply anaesthetized with chloral hydrate (0.4 g/kg bodyweight, Fluka Analytical, Buchs, Switzerland). Healthy controls were also anaesthetized in the same way.

**Fig. 1:**
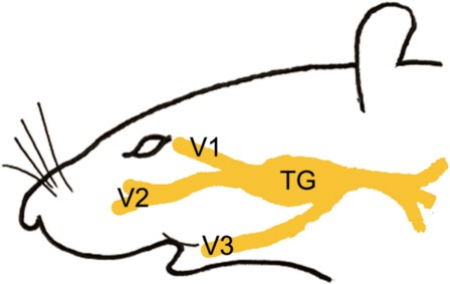
Schematic drawing of the rat head and the trigeminal ganglion (TG). The three branches V1, V2 and V3 of the trigeminal nerve are shown. Mainly the mandibular part (V3) of the TG was investigated

For immunohistochemistry, the rats were transcardially perfused with phosphate buffer for 5 min at a flow rate of 10 ml/min, followed by perfusion with 4 % paraformaldehyde in phosphate buffer for 20 min at the same flow rate. TGs from the right side were then dissected out, keeping the orientation of the three branches V1, V2 and V3 of the trigeminal nerve (Fig. 1). After overnight post-fixation, specimens were rinsed repeatedly in sucrose-enriched (10 %) Tyrode solution. The ganglia were frozen on dry ice and stored at −80 °C. The specimens were embedded in gelatin medium (30 % egg albumin, 3 % gelatin in distilled water), cryosectioned at 12 μm, mounted on Superfrost Plus coated slides (Menzel GmbH Co KG, Braunschweig, Germany) and stored at −20 °C until use.

For Western blot, TGs were harvested without fixation and frozen in liquid nitrogen. They were stored at −80 °C until use.

### Hematoxylin-eosin staining

For orientation of the three branches (Fig. 1) and examination of morphology and tissue condition, the sections were stained with Hematoxylin-Eosin (Htx-Eosin) using a standard protocol (Htx 4 min, water rinse, Eosin 30 s).

### Immunohistochemistry

Immunohistochemical demonstration of MAPKs (pERK1/2, pp38, pSAPK/JNK), NF-κB, CaMKII and DREAM was performed using indirect immunohistochemistry. CGRP, a major molecule in migraine therapy, and its receptor components CLR and RAMP1 were additionally immunohistochemically processed. Moreover, antibodies against inwardly rectifying potassium channel (Kir4.1, marker for glial cells) were used. Details of the primary and secondary antibodies used are given in Tables 1 and 2.

**Table 1:** Details of primary antibodies used for immunohistochemistry (IH) and Western blotting (WB)

Name	Product code	Host	Dilution		Source
			IH	WB	
Phospho-p44/42 MAPK (Erk1/2) (Thr202/Tyr204)	4376	Rabbit	1:50	1:1000	Cell Signaling Technology, Danvers, MA, USA
Phospho-p38 MAPK (Thr180/Tyr182)	9216	Mouse	1:400	1:2000	Cell Signaling Technology, Danvers, MA, USA
Phospho-SAPK/JNK (Thr183/Tyr185)	9255	Mouse	1:400	1:2000	Cell Signaling Technology, Danvers, MA, USA
Anti-NFκB p65 (phosphor S529)	ab97726	Rabbit	1:100	1:1000	Abcam; Cambridge, UK
CaMKII	ab52476	Rabbit	1:100	1:20000	Abcam; Cambridge, UK
DREAM (FL-214)	sc-9142	Rabbit	1:250	1:500	Santa Cruz Biotech, Santa Cruz, CA, USA
Anti-K_ir_4.1	APC-035	Rabbit	1:1000	--	Alomone Labs Ltd., Jerusalem, Israel
Anti-β-actin	Sc-47778	Mouse	--	1:5000	Santa Cruz Biotech, Santa Cruz, CA, USA
CGRP	ab81887	Mouse	1:100	--	Abcam; Cambridge, UK
CLR	132	Sheep	1:100	--	Merck & Co., Inc
RAMP1	844	Goat	1:100	--	Merck & Co., Inc

Abbreviations: *CaMKII* calcium calmodulin-dependent protein kinase II; *CGRP* calcitonin gene-related peptide; *CLR* calcitonin receptor-like receptor; *DREAM* downstream regulatory element antagonist modulator; *ERK1/2* extracellular signal-regulated kinase 1/2; *Kir4.1* inwardly rectifying potassium channel; *MAPK* mitogen-activated protein kinase; *NF-κB* nuclear factor kappa B; *RAMP1* receptor activity modifying protein 1; *SAPK/JNK* stress-activated protein kinase/c-jun N-terminal kinase

**Table 2:** Secondary antibodies used for immunohistochemistry (IH) and Western blotting (WB)

Conjugate and host	Against	Dilution	Source
IH			
FITC (goat)	Anti-rabbit	1:100	Cayman Chemical, Ann Arbor, MI, USA
Texas-Red (donkey)	Anti-rabbit	1:200	Jackson Immunoresearch, West Grove, PA, USA
DyLight 549 (donkey)	Anti-mouse	1:200	Jackson Immunoresearch, West Grove, PA, USA
DyLight 488 (donkey)	Anti-sheep	1:200	Jackson Immunoresearch, West Grove, PA, USA
Alexa 488 (donkey)	Anti-goat	1:400	Invitrogen, La Jolla, CA, USA
WB			
HRP-conjugated	Anti-rabbit	1:2000	Cell Signaling Technology, Danvers, MA, USA
HRP-conjugated	Anti-mouse	1:2000	Cell Signaling Technology, Danvers, MA, USA

Abbreviations: *FITC* fluorescein isothiocyanate; *HRP* horseradish peroxidase

Briefly, sections were rehydrated for 15 min in PBS containing 0.25 % Triton X-100 (PBS-T, Chemicon, Sweden). Sections were then exposed to primary antisera in PBS-T containing 1 % bovine serum albumin (BSA) overnight in a moist chamber at +4 °C. Thereafter, the sections were rinsed (PBS-T for 2 × 15 min), followed by incubation with secondary antibodies for 1 h in dark at room temperature. Sections were then rinsed in PBS-T for 3 × 10 min in room temperature and mounted with Vectashield (Vector Laboratories, Burlingame, CA, USA). Vectashield medium containing 4′, 6-diamino-2-phenylindole (DAPI, nucleus staining) was used.

Kir4.1 and pp38 or pSAPK/JNK, pERK1/2 and CLR or RAMP1, and NF-κB and CGRP double immunohistochemistry was additionally performed. The same protocol as described below was used during three consecutive days. (1) Day one: application of Kir4.1 (host rabbit); (2) Day two: application of secondary anti-rabbit antibodies and thereafter pp38 (host mouse); (3) Day three: application of secondary anti-mouse antibodies.

Omission of the primary antibody served as negative controls.

### Image analysis

Sections were examined and images were obtained using a light- and epifluorescence microscope (Nikon 80i, Tokyo, Japan) coupled to a Nikon DS-2 MV camera. FITC (480/30X), TRITC (540/24X) and DAPI (360/40X) filters were used (filter specifications are given in nanometers and X denotes excitation center wavelength/bandwidth). Adobe Photoshop CS3 (v.8.0, Adobe Systems, Mountain View, CA, USA) was used to visualize co-labeling by superimposing the digital images.

### Western blot

TG were homogenized in cell extract denaturing buffer (BioSource, USA) containing phosphatase and protease inhibitor cocktails (Sigma-Aldrich, Germany). After centrifugation (12,000 rpm, 4 °C, 10 min) the supernatants were collected. Protein concentrations were measured with a protein assay reagent (Bio-Rad Laboratories, Hercules, CA) and Tecan Infinite M200 microplate reader. Protein samples were mixed with Laemmli Sample Buffer (Bio-Rad Laboratories) and heated (95 °C, 4 min). Equal amounts (40 μg) of protein were loaded onto 4–15 % Ready Gel Precast Gels (Bio-Rad Laboratories) with a molecular weight marker (Precision Plus Protein Standard, Bio-Rad Laboratories). Gel electrophoresis was followed by blocking in 5 % non-fat milk or BSA and incubation with primary antibodies (4 °C, overnight), then with secondary antibodies (1 h, room temperature). Details of primary and secondary antibodies used are given in Tables 1 and 2. β-actin was used as an internal loading control. Finally, the membranes were incubated in enhanced chemiluminescent substrate (Pierce Protein Research Product, Thermo Scientific, Germany) and developed using KODAK light film and reagents (Sigma-Aldrich, Germany). The band optical density ratio was quantified using ImageJ software. Analysis of variance followed by Bonferroni (IBM SPSS Statistics, Version 20, 2011, Armonk, NY, USA) was used for statistical analysis (*p ≤ 0.05, **p ≤ 0.005).

## Results

### Hematoxylin-eosin

**Fig. 2:**
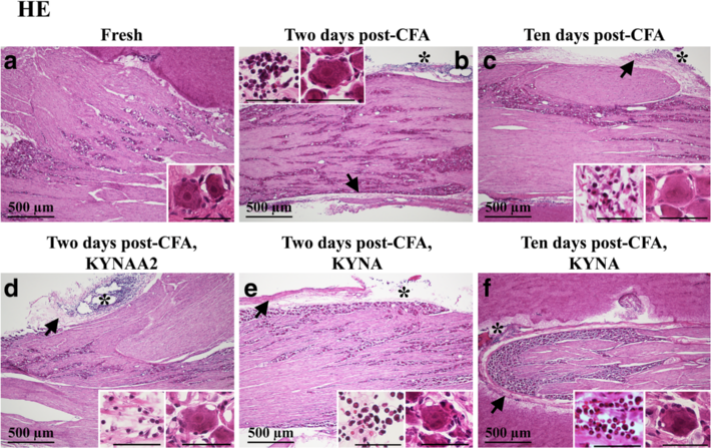
Hematoxylin-Eosin staining of trigeminal ganglia from control (fresh), inflammatory (2 or 10 days post-CFA) and treated (KYNA and KYNAA2) groups. **a** Neurons of different size, surrounded by SGCs, were found. **b**-**f** As a result of inflammation, perineurium thickening (arrows) and sterile abscess (asterisk) were present. Inserts show higher magnification of the neurons surrounded by SGCs. In b-f, the inserts show abscesses with polymorphonuclear cells and macrophages. Insert scale bars 50 μm

Most TGs displayed qualitatively good morphology as visualized with Htx-Eosin staining (Figs. 2a–f). Ganglia consisted of neurons enveloped by SGCs. Minor cell shrinkage was observed. In all TG following CFA injection into the TMJ, sterile abscesses containing polymorphonuclear cells and macrophages were observed (Fig. 2b–f). In addition, perineurium thickening was found in the CFA groups.

### Immunohistochemistry

The CFA induced inflammation showed main activation in the third division (Fig. 1, mandibular branch) of the TG, but some was also found in the rest of the ganglion. In our study, we primarily examined the changes present in the third division (data not shown).

#### pERK1/2

**Fig. 3:**
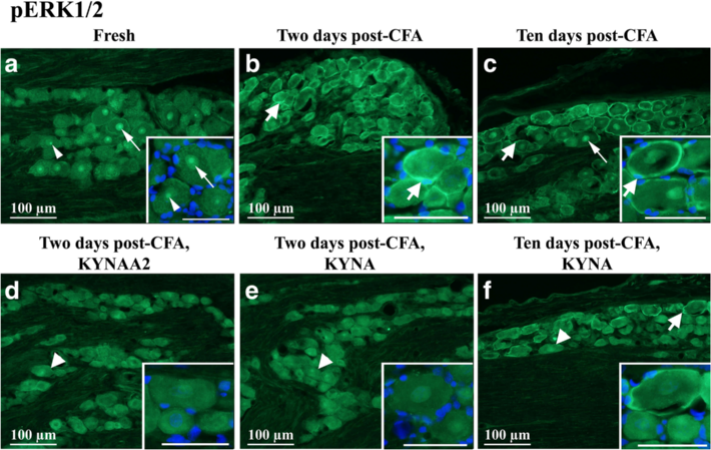
pERK1/2 immunohistochemistry of trigeminal ganglia from control (fresh), inflammatory (2 or 10 days post-CFA) and treated (KYNA and KYNAA2) groups. **a** In fresh trigeminal ganglia, pERK1/2 positive nuclei with nucleoli (thin arrows) and only nucleoli (thin arrow heads) were seen. **b**-**c** At 2 and 10 days post-CFA injection, pERK1/2-immunoreactive SGCs (thick arrows) were present. In addition, at 10 days post-CFA injection, some positive nuclei and nucleoli were also found (thin arrow). **d**-**e** KYNA and KYNAA2 abolished the pERK1/2-positivity in the SGCs (thick arrowheads) at 2 days. **f** After 10 days KYNA-treatment, both negative SGCs (thick arrowhead) and positive SGCs (thick arrow) were seen. Insert scale bars 50 μm

In fresh untreated TG, pERK1/2 immunoreactivity was detected in nuclei, including nucleoli, of the trigeminal neurons (Fig. 3a). In contrast, high-intensity pERK1/2 immunoreactivity was observed in SGCs at 2 days (Fig. 3b) and at 10 days post-CFA injection in the TMJ (Fig. 3c). In addition, increased pERK1/2 immunoreactivity was observed in neuronal nuclei and nucleoli at 10 days (Fig. 3c).

Treatment with KYNA and KYNAA2 abolished the pERK1/2 immunoreactivity in the SGCs at 2 days (Fig. 3d–e). The effect of KYNA-treatment was only partial at 10 days, with both pERK1/2-positive and pERK1/2-negative SGCs (Fig. 3f). pERK1/2 expression was not detected in the cytoplasm of neurons.

#### pp38

**Fig. 4:**
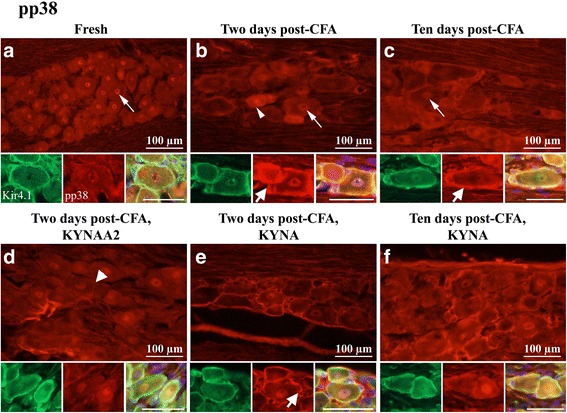
pp38 immunohistochemistry of trigeminal ganglia from control (fresh), inflammatory (2 or 10 days post-CFA) and treated (KYNA and KYNAA2) groups. **a** In fresh trigeminal ganglia, pp38 immunoreactive neuronal nuclei (thin arrow) were found. **b** At 2 days post-CFA injection, positive neuronal nuclei (thin arrow), a few neurons (thin arrowhead) and many SGCs (thick arrow) were present. **c** At 10 days, positive neuronal nuclei (thin arrow) and many SGCs (thick arrow) were found. In addition, some positive small- to medium sized neurons were found. **d** At 2 days post-CFA injection, KYNAA2 abolished the pp38-immunoreactivity in the SGCs (thick arrowheads) and in the small- and medium sized neurons. **e** At 2 days after KYNA-treatment many pp38-immunoreactive SGCs (thick arrow) were seen. No immunoreactivity was observed in the small- and medium sized neurons. **f** At 10 days, KYNA-treatment decreased the number and intensity of immunoreactive SGCs. Inserts show double staining with Kir4.1 (green) and pp38 (red) and the merged images with DAPI (nuclei staining), which revealed co-localization at 2 and 10 days post-CFA injection, and after KYNA-treatment at 2 and 10 days (b, c, e, f inserts). Insert scale bars 50 μm

In fresh TG, pp38 immunoreactivity was found in neuronal nuclei (Fig. 4a). At 2 and 10 days post-CFA injection, pp38 positive SGCs and neuronal nuclei were found, in addition to some stained small- to medium-sized neurons (Figs. 4b–c).

Both KYNA and KYNAA2 decreased the nuclei immunoreactivity at 2 days post-CFA injection. At 2 days KYNAA2, but not the KYNA treatment, reduced CFA-induced SGC immunoreactivity (Figs. 4d–e). At 10 days post-CFA injection, KYNA-treatment attenuated pp38 immunoreactivity in the SGCs but did not influence the pp38 immunoreactivity in the nuclei (Fig. 4f). To confirm that the SGCs were pp38 immunoreactive, double staining with the SGCs specific Kir4.1 was performed. The stainings revealed co-localization between pp38 and Kir4.1 in the SGCs (Figs. 4b, c, e, f inserts).

#### pSAPK/JNK

We found pSAPK/JNK immunoreactive SGCs in all six groups (Additional file 1: Figures S1a–f). At 2 days post-CFA injection, some homogenously stained neurons were seen in addition to the positive SGCs (Additional file 1: Figure S1b, arrow head). These immunoreactive neurons were not found in any of the other groups. To identify the SGC specificity, double staining with the SGC specific Kir4.1 was performed. Indeed, co-localization between pSAPK/JNK and Kir4.1 was seen (Additional file 1: Figures S1g-i).

#### NF-κB

**Fig. 5:**
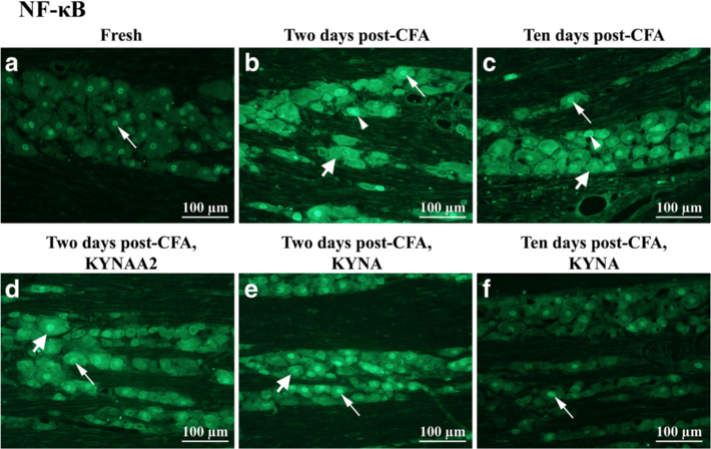
NF-κB staining of trigeminal ganglia from control (fresh), inflammatory (2 or 10 days post-CFA) and treated (KYNA and KYNAA2) groups. **a** In fresh trigeminal ganglia, NF-κB immunoreactive neuronal nuclei (thin arrow) were found. **b**-**c** At 2 and 10 days post-CFA injection, all of the neurons showed increased NF-κB intensity (thick arrow), out of them a few small- and medium-sized neurons seemed to be positive (thin arrowhead). Some of the neuronal nuclei showed higher NF-κB-expression (thin arrow). **d**-**e** KYNAA2- and KYNA-treatment at 2 days did not alter the expression of NF-κB; thick arrow points at intense neuronal NF-κB expression, some of these immunoreactive neurons were identified as small- to medium sized neurons (arrow head), thin arrow points at immunoreactive neuronal nuclei. **f** After 10 days KYNA-treatment, the staining was similar to the staining of the healthy group

NF-κB immunoreactive neuronal nuclei were found in fresh TG (Fig. 5a). At 2 and 10 days post-CFA injection, all neurons showed increased NF-κB immunoreactivity, both in the cytoplasm and in nuclei (Figs. 5b–c). At two days, KYNA- or KYNAA2-treatment did not alter the NF-κB neuronal immunoreactivity (Figs. 5d–e). At 10 days, KYNA-treatment abolished the presence of neuronal NF-κB immunoreactivity. The immunoreactivity was similar to fresh animals, suggesting a strong effect of KYNA at 10 days (Fig. 5f).

#### CaMKII

In fresh TG, the intensity of the staining varied from negative neurons to intense, homogenously stained CaMKII immunoreactive neurons of varying size (Additional file 2: Figure S2a). At 2 and 10 days post-CFA injection as well as in the KYNA and KYNAA2-treated groups, most of the neurons were immunoreactive (Additional file 2: Figures S2b–f). CaMKII expression was not detected in the SGCs or in the nuclei of the neurons.

#### DREAM

We found homogenous DREAM immunoreactivity in the cytoplasm of some neurons and in the neuronal nuclei of fresh TG (Additional file 3: Figure S3a). Two or 10 days post-CFA injection, inflammation resulted in increased DREAM expression in most of the neurons (Additional file 3: Figures S3b-c). KYNA- or KYNAA2-treatment did not show any changes in the expression (Additional file 3: Figures S3d–f).

**Fig. 6:**
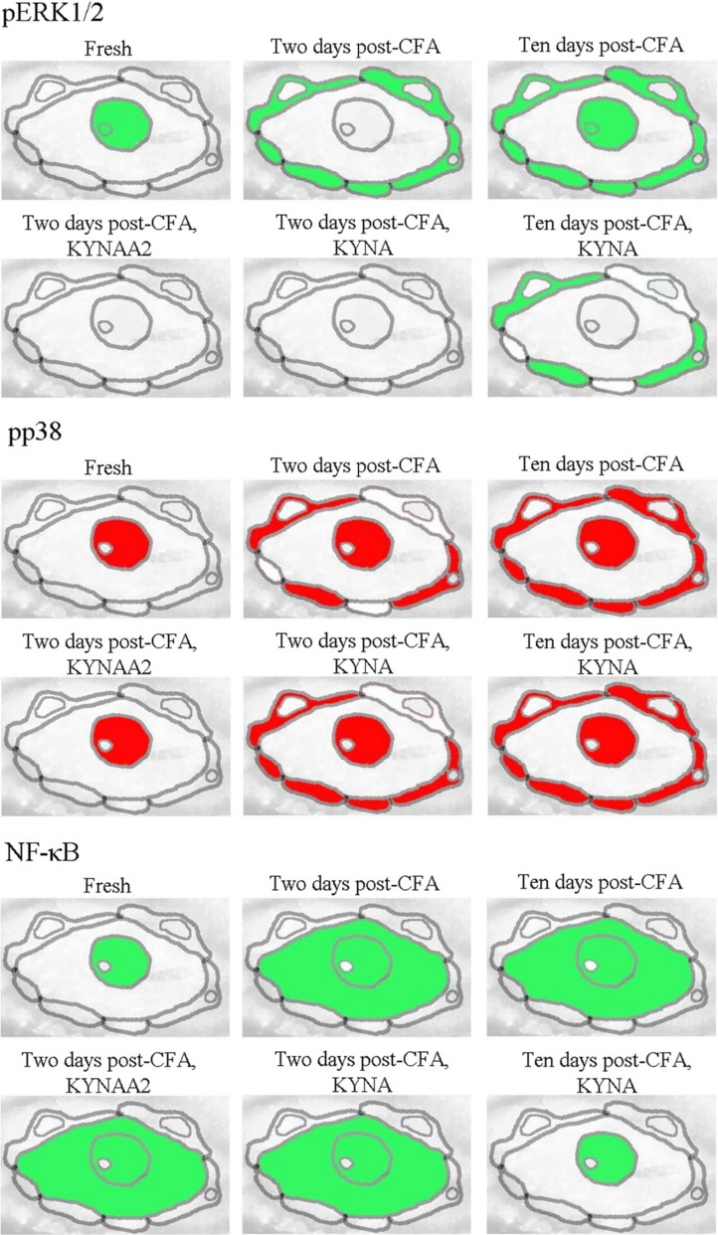
Schematic summary of pERK1/2, pp38 and NF-κB findings

Summary of pERK1/2, pp38, and NF-κB, immunohistochemistry results are schematically shown in Fig. 6.

### Double immunohistochemistry

#### pERK1/2 and CLR/RAMP1

**Fig. 7:**
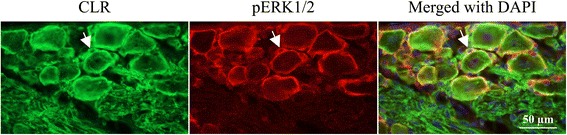
Double immunohistochemistry with pERK1/2 and CLR at 2 days post-CFA injection, which revealed co-localized pERK1/2 and CLR in the SGCs (arrows)

**Fig. 8:**
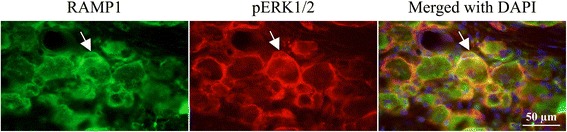
Double immunohistochemistry with pERK1/2 and RAMP1 at 2 days post-CFA injection, which revealed co-localized RAMP1 and pERK1/2 in the SGCs (arrows)

At two days post-CFA injection, double immunohistochemistry was performed with pERK1/2 and the CGRP receptor components CLR and RAMP1. We observed that pERK1/2 co-localized with CLR and RAMP1 in the SGCs (Figs. 7, 8).

#### NF-κB and CGRP

**Fig. 9:**
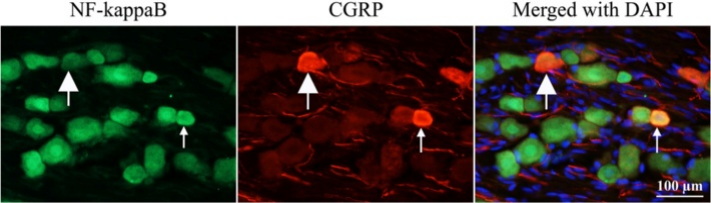
Double immunohistochemistry with NF-κB and CGRP at 2 days post-CFA injection. Thick arrowhead point at a CGRP-positive neuron, thin arrow at a double-stained CGRP and NF-κB neuron

Double immunohistochemistry with NF-κB and CGRP was carried out in the TG at 2 days post-CFA injection. In our hands, co-localization was found in some of the small- and medium sized neurons (Fig. 9).

#### Interactions

**Fig. 10:**
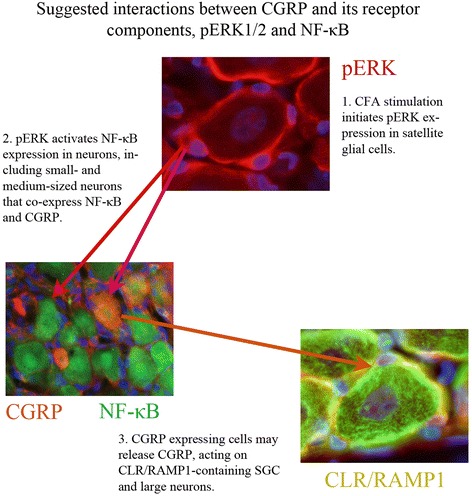
At 2 days post-CFA injection in the trigeminal ganglion the pERK1/2 immunoreactive SGCs induce the activation of NF-κB in the small- and medium sized neurons, which co-localize with CGRP. Presumably, the activation of NF-κB promotes the release of CGRP from small- and medium sized neurons. The released CGRP displays its effect on SGCs and large neurons, which contain the CGRP receptor components, the CLR and RAMP1 in the trigeminal ganglion

Figure 10 attempts to illustrate a suggested interaction between CGRP and its receptor components, pERK1/2 and NF-κB. At two days post-CFA injection in the trigeminal ganglion the pERK1/2 immunoreactive SGCs induce the activation of NF-κB in the small- and medium sized neurons, which co-localize with CGRP. Presumably, the activation of NF-κB promotes the release of CGRP from small-and medium sized neurons. The released CGRP displays its effect on SGCs and large neurons, which contain the CGRP receptor components CLR and RAMP1.

### Western blot

**Fig. 11:**
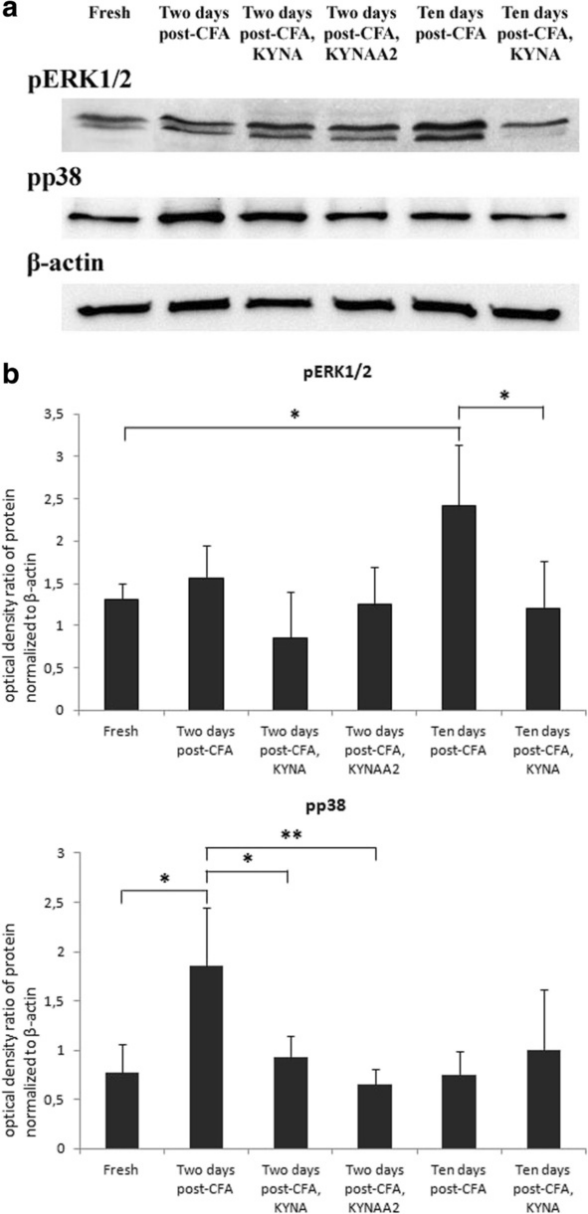
Western blot. **a** pERK1/2 and pp38 protein expression in rat trigeminal ganglion in healthy conditions, at 2 and 10 days post-CFA injection and after treatment with KYNA or KYNAA2. The protein levels were examined by Western blot. **b** Graphical representation of optical density ratio of pERK1/2 and pp38 protein normalized to β-actin. Values are mean ± S.D. (*n =* 6) (*p ≤ 0.05, **p ≤ 0.005)

The protein level of pERK1/2, pp38, pSAPK/JNK, CaMKII, NF-κB and DREAM in TG was measured by Western blot. Densitometric analyses confirmed that pERK1/2 was significantly enhanced at10 days post-CFA injection (*p =* 0.016). This level was normalized in the 10 days KYNA treated animals (*p =* 0.008) (Fig. 11). There was a significant increase observed at 2 days post-CFA injection in the pp38 level (*p =* 0.011). This level of phosphorylated p38 was significantly lower in animals treated with KYNA (*p =* 0.027) or KYNAA2 (*p =* 0.004) two days after the CFA injection (Fig. 11). There were no significant changes in pSAPK/JNK, NF-κB, CaMKII and DREAM protein levels (data not shown). Levels of β-actin confirmed equal loading of protein samples.

## Discussion

This is the first study that examines in details the effects of KYNA and KYNAA2 on the expressional alterations in inflammation-related molecules in the mandibular part of the TG (Fig. 1) after administration of CFA into the TMJ. We found at day 2 and at day 10 post-CFA injection, an increased expression of pERK1/2 and pp38 in neurons or SGCs, while only neurons showed an increased NF-κB and CaMKII immunoreactivity. Treatment with the glutamate modulators KYNA and its derivate KYNAA2 reduced or normalized the pERK1/2, pp38 and NF-κB immunoreactivity. The findings suggest that local inflammation of the TMJ, induced by CFA, results in an inflammation response in the TG where the TMJ sensory fibers have their cell bodies. Interestingly, this involves both neurons and SGCs which represent together an anatomical (Fig. 2a, insert) and functional unit [46].

It has been already demonstrated, using SGC detecting glial fibrillary acidic protein (GFAP) immunohistochemistry that 24 and 72 h post-saline injections into TMJ have the same inflammatory effect as the ones seen in the contralateral CFA injected TMJ tissue, i.e. few GFAP immunoreactive SGCs. On the other hand, 24 and 72 h post-CFA treated ipsilateral side showed a significant increase of GFAP immunoreactive SGCs [42]. Moreover, similar results were found during the measurement of the Evans’ Blue dye extravasation in the TMJ. Saline injection into TMJ resulted in the same inflammatory effect as was found in the contralateral side of the CFA-injection. The ipsilateral TMJ displayed 5–8 times higher Evans’ Blue concentration compared to saline injected animals [42]. For these reasons, we used fresh animals as controls in order to achieve the normal protein expression and to compare this expression to the CFA-injected ipsilateral side, and furthermore to evaluate possible effects of KYYNA and KYNAA2 treatments.

The CFA injection method is a widely accepted inflammatory model of the trigeminal activation [47]. The proposed mechanism of this method is that after the CFA stimulation the number of macrophages increases dramatically in the TG around the neurons [48], which release cytokines, MAPKs and other mediators [47]. The increased release of MAPK signaling proteins takes part in the process of the peripheral sensitization of the primary nociceptive neurons in the sensory ganglia [10, 47, 49].

### pERK1/2, pp38 and pSAPK/JNK

SGCs form a tight envelope around neurons (Fig. 2a), coupled by gap junctions, in sensory ganglia. Many studies have investigated SGC morphology and physiology, giving the subject for numerous reviews [1, 2, 50–54]. The expression of Kir4.1, glutamate recycling component, the glutamate-aspartate transporter and P2Y4 metabotropic purinergic receptors have been shown in SGCs in the TG [55]. Silencing the Kir4.1 expression in the TG results in spontaneous and evoked pain sensation, suggesting its importance in pain [56]. Increase in SGCs S100B expression 2 h after capsaicin injection into the TMJ and activation of p38 after NO-proton or TNF-α stimulation have been demonstrated [17, 57, 58]. Garrett and Durham [59] injected CFA or capsaicin into the TMJ, which resulted in increased protein expression of connexin26 in the SGCs in the TG. Cady et al. [60] showed pERK1/2 activation in the SGCs 2–24 h after CGRP injection into the TMJ. In the present study we show the time-related activation of MAPKs (pERK1/2, pp38) in SGCs post-CFA injection, which is diminished after KYNA and/or KYNAA2 treatment (Figs. 3 and 4). Together, these data suggest that SGCs play a role in spontaneous and evoked pain sensation, and inflammation-induced peripheral sensitization in the TG.

To confirm results obtained from immunohistochemistry, Western blot was used. Analyses confirmed that pERK1/2 was significantly enhanced at 10 days post-CFA injection (*p =* 0.016). This level was normalized in the 10 days KYNA treated animals (*p =* 0.008) (Fig. 11). In addition, there was a significant increase observed at 2 days post-CFA injection in the pp38 level (*p =* 0.011). This level of phosphorylated p38 was significantly lower in animals treated with KYNA (*p =* 0.027) or KYNAA2 (*p =* 0.004) two days after the CFA injection (Fig. 11).

### NF-κB, CaMKII and DREAM

The MAPKs are activated by inflammatory mediators and initiate the expression of transcription factors, such as NF-κB [19–21], while CaMKII and DREAM are involved in nociception and pain transmission [24, 25, 27, 28]. Our results showed a strong NF-κB activation in the neuronal cytoplasm, both 2 and 10 days after CFA-stimulation (Figs. 5b–c). This suggests that the regulation of transcription factor is a time-consuming process. In this inflammatory model no sharp differences were observed in the glial or neuronal expression of CaMKII and DREAM (Additional files 2 and 3: Figures S2-3). The stimulation by inflammatory mediators at the terminals of unmyelinated nerve fibers results in activation of MAPKs and transcription factors in the trigeminal system.

### CGRP

Previous studies suggest that glutamate is present in the trigeminovascular system [61–63]. Eftekhari et al. has demonstrated that glutamate and CGRP are mostly expressed in separate neurons in the rat and rhesus TG [64]. This suggests that the trigeminal neurons that express CGRP do not contain glutamate as a co-transmitter. Furthermore, in a double-staining of glutamate and CGRP receptor components, the CLR or the RAMP1 showed a co-localization with glutamate in the trigeminal neurons of rat and rhesus [64]. This opens up the possibility of interaction between the glutamate system and CGRP receptors.

At two days post-CFA injection in the TG the pERK1/2 immunoreactive SGCs induce the activation of NF-κB in the small- and medium sized neurons, which co-localize with CGRP. Presumably, the activation of NF-κB promotes the release of CGRP from small- and medium sized neurons. The released CGRP displays its effect on SGCs and large neurons, which contain the CGRP receptor components, the CLR and RAMP1 in the TG (Fig. 10). As CLR and RAMP1 co-localize with pERK1/2 in the SGCs (Fig. 7–8), this process may cause a self-amplifying cycle. These observations and the raised hypothesis initiate the need of further investigations.

In TMJ inflammation, there is a varying degree of increase in the concentrations of different inflammatory mediators (such as CGRP, nerve growth factor, interleukin-1β and TNF-α) in the TG [4]. CFA stimulation of the TMJ results in a significantly higher trigeminal Fos protein expression than the stimulation of the perioral skin as there is heavy innervation of the TMJ by unmyelinated nerve endings [65]. An immunohistochemical study revealed that the NR1 subunit of the NMDA receptor is expressed in all sized of the neurons in the TG, while the NR2 subunit is mainly in the small sized neurons and in the SGCs [5]. It has been also reported that NMDA receptor antagonists (DL-2-amino-5-phosphonovaleric acid and Ifenprodil) blocked CFA-induced mechanical hypersensitivity of the TMJ region and the constitutive expression of NMDA receptor subunits in the TG [5].

The kynurenine pathway of tryptophan metabolism produces several neuroactive by-products (such as KYNA, xanthurenic acid, cinnabarinic acid and quinolinic acid), acting on both NMDA and metabotropic glutamate receptors [37, 66]. KYNA is an endogenous excitatory amino acid receptor blocker. KYNA acts as a potent NMDA receptor antagonist, as its primary site of action is the strychnine-insensitive glycine site of the NMDA receptor at low micromolar concentrations [36]. Hilmas et al. [38] demonstrated that KYNA non-competitively and voltage independently inhibits alpha7 nicotinic acetylcholine receptors located on pre-synaptic terminals. In addition KYNA is a weak amino-3-hydroxy-5-methyl-4-isoxazolepropionic acid (AMPA) or kainate sensitive glutamate receptor blocker in micromolar to millimolar concentrations, while it is able to facilitate AMPA receptors in nanomolar concentrations [39, 40]. Our findings may suggest a function of KYNA and KYNAA2, in the used doses, in glutamatergic and nicotinergic neurotransmission.

## Conclusions

The CFA-induced inflammatory model for the TG activation provided a time-related expression of MAPK (pERK1/2; pp38) and NF-κB. It involves both the neuronal and glial activation, which points the possible neuron-glia interaction during this process. The administration of the endogenous NMDA-receptor antagonists, KYNA and its derivative KYNAA2, resulted in the inhibition of the induced signaling system of the TG, which further points the importance of the glutamate receptors in this mechanism.

## Additional files

Additional file 1: Figure S1. pSAPK/JNK immunohistochemistry of trigeminal ganglia from control (fresh), inflammatory (2 or 10 days post-CFA) and treated (KYNA and KYNAA2) groups. a-f pSAPK/JNK immunoreactive SGCs were found in all six groups. No difference was noticed between the groups except for some homogenously stained neurons at 2 days post-CFA (b). g-i To identify the SGC specificity, double staining with the SGC specific Kir4.1 was performed. Indeed, co-localization between pSAPK/JNK and Kir4.1 was seen. Insert scale bars 50 μm. (JPG 10261 kb) Additional file 2: Figure S2. CaMKII immunohistochemistry of trigeminal ganglia from control (fresh), inflammatory (2 or 10 days post-CFA) and treated (KYNA and KYNAA2) groups. a In fresh trigeminal ganglia, the intensity of the staining varied from negative neurons to intense, homogenously stained CaMKII immunoreactive neurons of varying size (thin arrowhead - intensely stained neuron; thick arrow - neurons with lesser expression; thin arrow - negative neurons). b-f At 2 and 10 days post-CFA injection as well as in the KYNA and KYNAA2-treated groups, most of the neurons were immunoreactive. CaMKII expression was not detected in the SGCs or in the nuclei of the neurons. Insert scale bars 50 μm. (JPG 4521 kb) Additional file 3: Figure S3. DREAM immunohistochemistry of trigeminal ganglia from control (fresh), inflammatory (2 or 10 days post-CFA) and treated (KYNA and KYNAA2) groups. a In fresh trigeminal ganglia homogenous DREAM immunoreactivity was found in the cytoplasm of some neurons (arrow head) and in the neuronal nuclei (thin arrow). b-c Two or 10 days post-CFA injection, inflammation resulted in increased DREAM expression in most of the neurons (arrow head). d-f KYNA- or KYNAA2-treatment did not show any changes in the expression. Insert scale bars 50 μm. (JPG 5002 kb)

## Abbreviations

AMPA: Amino-3-hydroxy-5-methyl-4-isoxazolepropionic acid
BBB: Blood–brain barrier
CaMKII: Calcium calmodulin-dependent protein kinase II
CFA: Complete Freund’s adjuvant
CGRP: Calcitonin gene-related peptide
CLR: Calcitonin receptor-like receptor
DREAM: Downstream regulatory element antagonist modulator
ERK1/2: Extracellular signal-regulated kinase 1/2
GFAP: Glial fibrillary acidic protein
Kir4.1: Inwardly rectifying potassium channel
KYNA: Kynurenic acid
KYNAA2: Kynurenic acid amide 2, *N*-(2-*N*,*N*-dimethylaminoethyl)-4-oxo-1*H*-quinoline-2-carboxamide hydrochloride
MAPKs: Mitogen-activated protein kinases
NF-κB: Nuclear factor kappa B
NMDA: N-methyl-D-aspartate receptor
NO: Nitric oxide
RAMP1: Receptor activity modifying protein 1
SAPK/JNK: Stress-activated protein kinase/c-jun N-terminal kinase
SGC: Satellite glial cell
TG: Trigeminal ganglion
TNF-α: Tumor necrosis factor-α
TMJ: Temporomandibular joint

## References

[1] Hanani M (2005). Satellite glial cells in sensory ganglia: from form to function. Brain Res Brain Res Rev.

[2] Hanani M (2010). Satellite glial cells in sympathetic and parasympathetic ganglia: in search of function. Brain Res Rev.

[3] Tajti J, Kuris A, Vecsei L, Xu CB, Edvinsson L (2011). Organ culture of the trigeminal ganglion induces enhanced expression of calcitonin gene-related peptide via activation of extracellular signal-regulated protein kinase 1/2. Cephalalgia.

[4] Hutchins B, Spears R, Hinton RJ, Harper RP (2000). Calcitonin gene-related peptide and substance P immunoreactivity in rat trigeminal ganglia and brainstem following adjuvant-induced inflammation of the temporomandibular joint. Arch Oral Biol.

[5] Ivanusic JJ, Beaini D, Hatch RJ, Staikopoulos V, Sessle BJ, Jennings EA (2011). Peripheral N-methyl-d-aspartate receptors contribute to mechanical hypersensitivity in a rat model of inflammatory temporomandibular joint pain. Eur J Pain.

[6] Spears R, Dees LA, Sapozhnikov M, Bellinger LL, Hutchins B (2005). Temporal changes in inflammatory mediator concentrations in an adjuvant model of temporomandibular joint inflammation. J Orofac Pain.

[7] Cady RJ, Durham PL (2010). Cocoa-enriched diets enhance expression of phosphatases and decrease expression of inflammatory molecules in trigeminal ganglion neurons. Brain Res.

[8] Cady RJ, Hirst JJ, Durham PL (2010). Dietary grape seed polyphenols repress neuron and glia activation in trigeminal ganglion and trigeminal nucleus caudalis. Mol Pain.

[9] Wang S, Lim G, Mao J, Sung B (2009). Regulation of the trigeminal NR1 subunit expression induced by inflammation of the temporomandibular joint region in rats. Pain.

[10] Ji RR (2004). Peripheral and central mechanisms of inflammatory pain, with emphasis on MAP kinases. Curr Drug Targets Inflamm Allergy.

[11] Kingwell K (2011). Pain: MAPK inhibitor shows promise in clinical trial for neuropathic pain. Nat Rev Neurol.

[12] Boulton TG, Nye SH, Robbins DJ, Ip NY, Radziejewska E, Morgenbesser SD (1991). ERKs: a family of protein-serine/threonine kinases that are activated and tyrosine phosphorylated in response to insulin and NGF. Cell.

[13] Derijard B, Hibi M, Wu IH, Barrett T, Su B, Deng T (1994). JNK1: a protein kinase stimulated by UV light and Ha-Ras that binds and phosphorylates the c-Jun activation domain. Cell.

[14] Kyriakis JM, Banerjee P, Nikolakaki E, Dai T, Rubie EA, Ahmad MF, et al (1994) The stress-activated protein kinase subfamily of c-Jun kinases. Nature 369:156–160 10.1038/369156a0 8177321

[15] Raingeaud J, Gupta S, Rogers JS, Dickens M, Han J, Ulevitch RJ, et al (1995) Pro-inflammatory cytokines and environmental stress cause p38 mitogen-activated protein kinase activation by dual phosphorylation on tyrosine and threonine. J Biol Chem 270:7420–7426 10.1074/jbc.270.13.7420 7535770

[16] Lewis TS, Shapiro PS, Ahn NG (1998). Signal transduction through MAP kinase cascades. Adv Cancer Res.

[17] Bowen EJ, Schmidt TW, Firm CS, Russo AF, Durham PL (2006). Tumor necrosis factor-alpha stimulation of calcitonin gene-related peptide expression and secretion from rat trigeminal ganglion neurons. J Neurochem.

[18] Cowan KJ, Storey KB (2003). Mitogen-activated protein kinases: new signaling pathways functioning in cellular responses to environmental stress. J Exp Biol.

[19] Schramek H (2002). MAP kinases: from intracellular signals to physiology and disease. News Physiol Sci.

[20] Barnes PJ, Karin M (1997). Nuclear factor-kappaB: a pivotal transcription factor in chronic inflammatory diseases. N Engl J Med.

[21] Buchanan MM, Hutchinson M, Watkins LR, Yin H (2010). Toll-like receptor 4 in CNS pathologies. J Neurochem.

[22] Park HJ, Park OJ, Shin J (2005). Receptor activator of NF-kappaB ligand enhances the activity of macrophages as antigen presenting cells. Exp Mol Med.

[23] Lee MK, Han SR, Park MK, Kim MJ, Bae YC, Kim SK (2011). Behavioral evidence for the differential regulation of p-p38 MAPK and p-NF-kappaB in rats with trigeminal neuropathic pain. Mol Pain.

[24] Fang L, Wu J, Lin Q, Willis WD (2002). Calcium-calmodulin-dependent protein kinase II contributes to spinal cord central sensitization. J Neurosci.

[25] Ogawa A, Dai Y, Yamanaka H, Iwata K, Niwa H, Noguchi K (2005). Ca(2+)/calmodulin-protein kinase IIalpha in the trigeminal subnucleus caudalis contributes to neuropathic pain following inferior alveolar nerve transection. Exp Neurol.

[26] Vamos E, Fejes A, Koch J, Tajti J, Fulop F, Toldi J (2010). Kynurenate derivative attenuates the nitroglycerin-induced CamKIIalpha and CGRP expression changes. Headache.

[27] Carrion AM, Link WA, Ledo F, Mellstrom B, Naranjo JR (1999). DREAM is a Ca2 + −regulated transcriptional repressor. Nature.

[28] Zaidi NF, Thomson EE, Choi EK, Buxbaum JD, Wasco W (2004). Intracellular calcium modulates the nuclear translocation of calsenilin. J Neurochem.

[29] Cheng HY, Pitcher GM, Laviolette SR, Whishaw IQ, Tong KI, Kockeritz LK (2002). DREAM is a critical transcriptional repressor for pain modulation. Cell.

[30] Ho TW, Edvinsson L, Goadsby PJ (2010). CGRP and its receptors provide new insights into migraine pathophysiology. Nat Rev Neurol.

[31] Eftekhari S, Salvatore CA, Calamari A, Kane SA, Tajti J, Edvinsson L (2010). Differential distribution of calcitonin gene-related peptide and its receptor components in the human trigeminal ganglion. Neuroscience.

[32] Edvinsson L (2008). CGRP blockers in migraine therapy: where do they act?. Br J Pharmacol.

[33] Giles GI, Collins CA, Stone TW, Jacob C (2003). Electrochemical and in vitro evaluation of the redox-properties of kynurenine species. Biochem Biophys Res Commun.

[34] Vecsei L, Szalardy L, Fulop F, Toldi J (2013). Kynurenines in the CNS: recent advances and new questions. Nat Rev Drug Discov.

[35] Fulop F, Szatmari I, Toldi J, Vecsei L (2012). Modifications on the carboxylic function of kynurenic acid. J Neural Transm.

[36] Birch PJ, Grossman CJ, Hayes AG (1988). Kynurenic acid antagonises responses to NMDA via an action at the strychnine-insensitive glycine receptor. Eur J Pharmacol.

[37] Ganong AH, Cotman CW (1986). Kynurenic acid and quinolinic acid act at N-methyl-D-aspartate receptors in the rat hippocampus. J Pharmacol Exp Ther.

[38] Hilmas C, Pereira EF, Alkondon M, Rassoulpour A, Schwarcz R, Albuquerque EX (2001). The brain metabolite kynurenic acid inhibits alpha7 nicotinic receptor activity and increases non-alpha7 nicotinic receptor expression: physiopathological implications. J Neurosci.

[39] Prescott C, Weeks AM, Staley KJ, Partin KM (2006). Kynurenic acid has a dual action on AMPA receptor responses. Neurosci Lett.

[40] Rozsa E, Robotka H, Vecsei L, Toldi J (2008). The Janus-face kynurenic acid. J Neural Transm.

[41] Kameoka S, Matsumoto K, Kai Y, Yonehara Y, Arai Y, Honda K (2010). Establishment of temporomandibular joint puncture technique in rats using in vivo micro-computed tomography (R_mCT(R)). Dentomaxillofac Radiol.

[42] Villa G, Ceruti S, Zanardelli M, Magni G, Jasmin L, Ohara PT, et al (2010) Temporomandibular joint inflammation activates glial and immune cells in both the trigeminal ganglia and in the spinal trigeminal nucleus. Mol Pain 6:89 10.1186/1744-8069-6-89 PMC3017032 21143950

[43] Knyihar-Csillik E, Mihaly A, Krisztin-Peva B, Robotka H, Szatmari I, Fulop F (2008). The kynurenate analog SZR-72 prevents the nitroglycerol-induced increase of c-fos immunoreactivity in the rat caudal trigeminal nucleus: comparative studies of the effects of SZR-72 and kynurenic acid. Neurosci Res.

[44] Vamos E, Pardutz A, Varga H, Bohar Z, Tajti J, Fulop F (2009). l-kynurenine combined with probenecid and the novel synthetic kynurenic acid derivative attenuate nitroglycerin-induced nNOS in the rat caudal trigeminal nucleus. Neuropharmacology.

[45] Zhou Q, Imbe H, Dubner R, Ren K (1999). Persistent Fos protein expression after orofacial deep or cutaneous tissue inflammation in rats: implications for persistent orofacial pain. J Comp Neurol.

[46] Dublin P, Hanani M (2007). Satellite glial cells in sensory ganglia: their possible contribution to inflammatory pain. Brain Behav Immun.

[47] Cady RJ, Denson JE, Sullivan LQ, Durham PL (2014). Dual orexin receptor antagonist 12 inhibits expression of proteins in neurons and glia implicated in peripheral and central sensitization. Neuroscience.

[48] Nakamura R, Nishimura T, Ochiai T, Nakada S, Nagatani M, Ogasawara H (2013). Availability of a microglia and macrophage marker, iba-1, for differential diagnosis of spontaneous malignant reticuloses from astrocytomas in rats. J Toxicol Pathol.

[49] Dai Y, Iwata K, Fukuoka T, Kondo E, Tokunaga A, Yamanaka H (2002). Phosphorylation of extracellular signal-regulated kinase in primary afferent neurons by noxious stimuli and its involvement in peripheral sensitization. J Neurosci.

[50] Chiang CY, Dostrovsky JO, Iwata K, Sessle BJ (2011). Role of glia in orofacial pain. Neuroscientist.

[51] Gosselin RD, Suter MR, Ji RR, Decosterd I (2010). Glial cells and chronic pain. Neuroscientist.

[52] Ohara PT, Vit JP, Bhargava A, Romero M, Sundberg C, Charles AC (2009). Gliopathic pain: when satellite glial cells go bad. Neuroscientist.

[53] Scholz J, Woolf CJ (2007). The neuropathic pain triad: neurons, immune cells and glia. Nat Neurosci.

[54] Takeda M, Takahashi M, Matsumoto S (2009). Contribution of the activation of satellite glia in sensory ganglia to pathological pain. Neurosci Biobehav Rev.

[55] Vit JP, Jasmin L, Bhargava A, Ohara PT (2006). Satellite glial cells in the trigeminal ganglion as a determinant of orofacial neuropathic pain. Neuron Glia Biol.

[56] Vit JP, Ohara PT, Bhargava A, Kelley K, Jasmin L (2008). Silencing the Kir4.1 potassium channel subunit in satellite glial cells of the rat trigeminal ganglion results in pain-like behavior in the absence of nerve injury. J Neurosci.

[57] Thalakoti S, Patil VV, Damodaram S, Vause CV, Langford LE, Freeman SE (2007). Neuron-glia signaling in trigeminal ganglion: implications for migraine pathology. Headache.

[58] Freeman SE, Patil VV, Durham PL (2008). Nitric oxide-proton stimulation of trigeminal ganglion neurons increases mitogen-activated protein kinase and phosphatase expression in neurons and satellite glial cells. Neuroscience.

[59] Garrett FG, Durham PL (2008). Differential expression of connexins in trigeminal ganglion neurons and satellite glial cells in response to chronic or acute joint inflammation. Neuron Glia Biol.

[60] Cady RJ, Glenn JR, Smith KM, Durham PL (2011). Calcitonin gene-related peptide promotes cellular changes in trigeminal neurons and glia implicated in peripheral and central sensitization. Mol Pain.

[61] Ge SN, Ma YF, Hioki H, Wei YY, Kaneko T, Mizuno N (2010). Coexpression of VGLUT1 and VGLUT2 in trigeminothalamic projection neurons in the principal sensory trigeminal nucleus of the rat. J Comp Neurol.

[62] Hegarty DM, Tonsfeldt K, Hermes SM, Helfand H, Aicher SA (2010). Differential localization of vesicular glutamate transporters and peptides in corneal afferents to trigeminal nucleus caudalis. J Comp Neurol.

[63] Li JL, Xiong KH, Dong YL, Fujiyama F, Kaneko T, Mizuno N (2003). Vesicular glutamate transporters, VGluT1 and VGluT2, in the trigeminal ganglion neurons of the rat, with special reference to coexpression. J Comp Neurol.

[64] Eftekhari S, Salvatore CA, Johansson S, Chen TB, Zeng Z, Edvinsson L (2015) Localization of CGRP, CGRP receptor, PACAP and glutamate in trigeminal ganglion. Relation to the blood–brain barrier. Brain Res 1600:93-109 10.1016/j.brainres.2014.11.031 25463029

[65] Imbe H, Iwata K, Zhou QQ, Zou S, Dubner R, Ren K (2001). Orofacial deep and cutaneous tissue inflammation and trigeminal neuronal activation. Implications for persistent temporomandibular pain. Cells Tissues Organs.

[66] Curto M, Lionetto L, Fazio F, Mitsikostas DD, Martelletti P (2015). Fathoming the kynurenine pathway in migraine: why understanding the enzymatic cascades is still critically important. Intern Emerg Med.

