# Supplementary figures and images for: Kynurenic acid modulates experimentally induced inflammation in the trigeminal ganglion
Source: J Headache Pain. 2015 Dec 1;16:99. doi: 10.1186/s10194-015-0581-x (PMC4666855; doi:10.1186/s10194-015-0581-x)

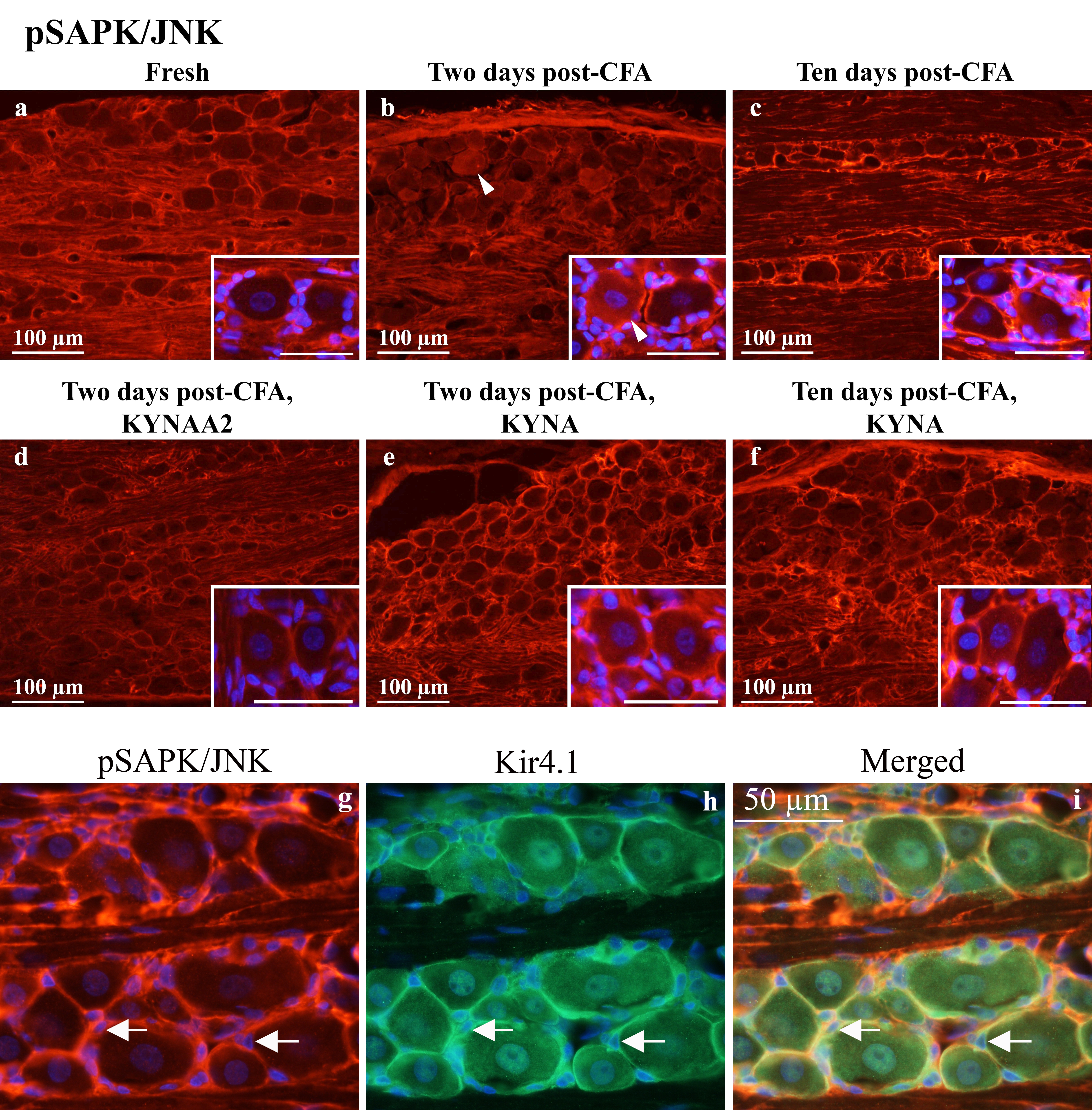

Supplement: Additional file 1: Figure S1. — pSAPK/JNK immunohistochemistry of trigeminal ganglia from control (fresh), inflammatory (2 or 10 days post-CFA) and treated (KYNA and KYNAA2) groups. a-f pSAPK/JNK immunoreactive SGCs were found in all six groups. No difference was noticed between the groups except for some homogenously stained neurons at 2 days post-CFA (b). g-i To identify the SGC specificity, double staining with the SGC specific Kir4.1 was performed. Indeed, co-localization between pSAPK/JNK and Kir4.1 was seen. Insert scale bars 50 μm. (JPG 10261 kb) [file 10194_2015_581_MOESM1_ESM.jpg]

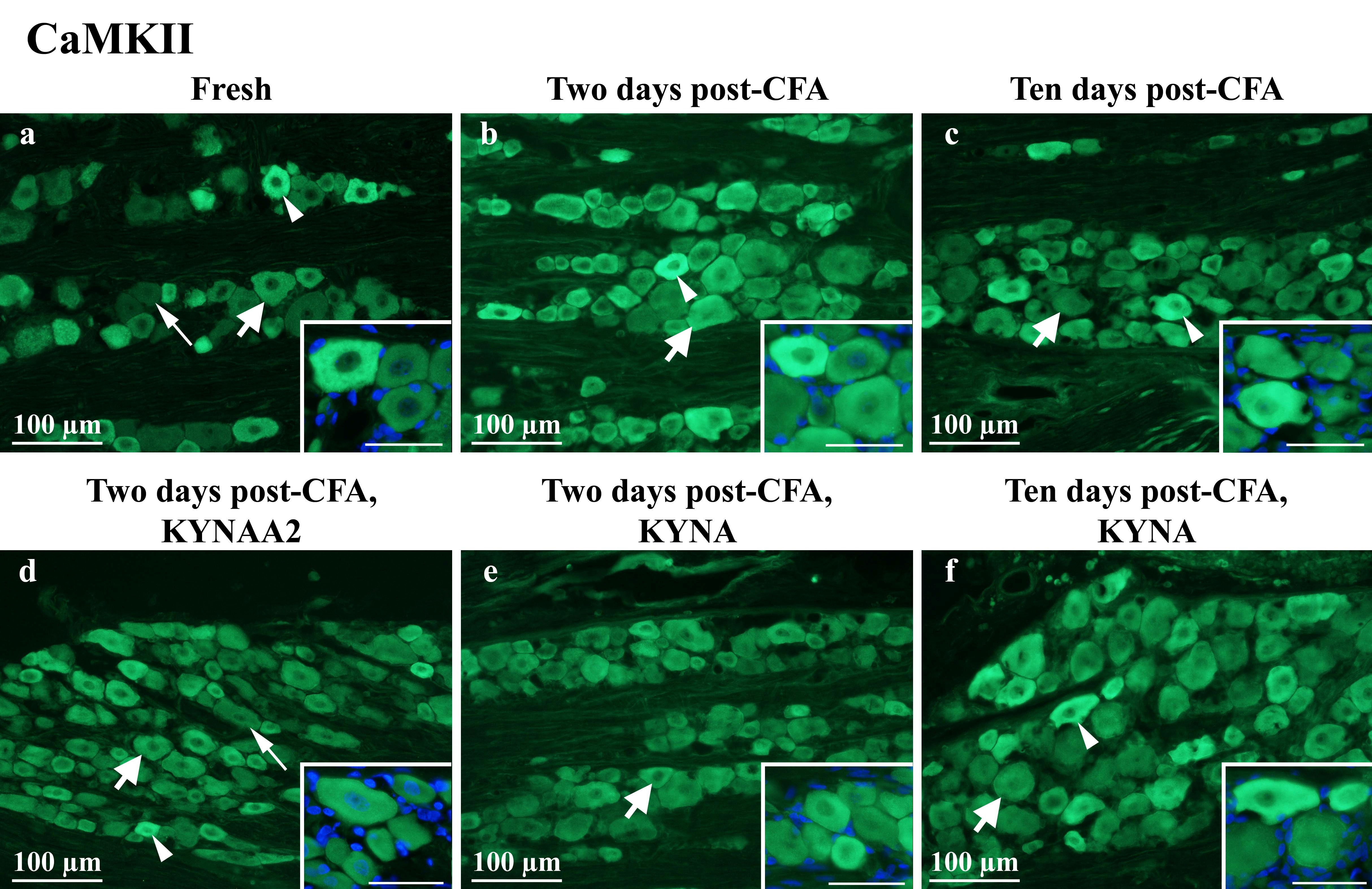

Supplement: Additional file 2: Figure S2. — CaMKII immunohistochemistry of trigeminal ganglia from control (fresh), inflammatory (2 or 10 days post-CFA) and treated (KYNA and KYNAA2) groups. a In fresh trigeminal ganglia, the intensity of the staining varied from negative neurons to intense, homogenously stained CaMKII immunoreactive neurons of varying size (thin arrowhead - intensely stained neuron; thick arrow - neurons with lesser expression; thin arrow - negative neurons). b-f At 2 and 10 days post-CFA injection as well as in the KYNA and KYNAA2-treated groups, most of the neurons were immunoreactive. CaMKII expression was not detected in the SGCs or in the nuclei of the neurons. Insert scale bars 50 μm. (JPG 4521 kb) [file 10194_2015_581_MOESM2_ESM.jpg]

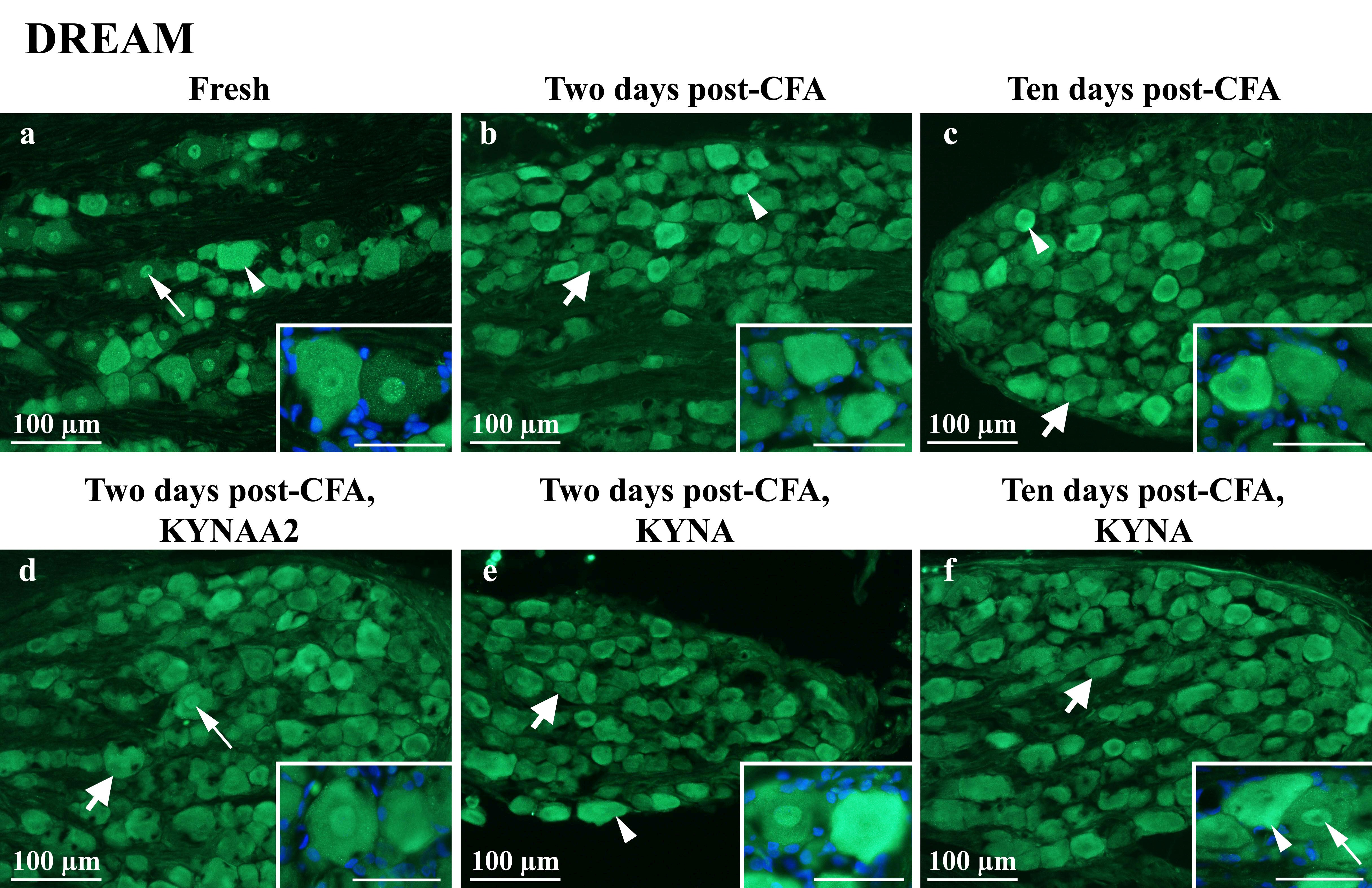

Supplement: Additional file 3: Figure S3. — DREAM immunohistochemistry of trigeminal ganglia from control (fresh), inflammatory (2 or 10 days post-CFA) and treated (KYNA and KYNAA2) groups. a In fresh trigeminal ganglia homogenous DREAM immunoreactivity was found in the cytoplasm of some neurons (arrow head) and in the neuronal nuclei (thin arrow). b-c Two or 10 days post-CFA injection, inflammation resulted in increased DREAM expression in most of the neurons (arrow head). d-f KYNA- or KYNAA2-treatment did not show any changes in the expression. Insert scale bars 50 μm. (JPG 5002 kb) [file 10194_2015_581_MOESM3_ESM.jpg]
